# The low-copy nuclear gene *Agt1* as a novel DNA barcoding marker for Bromeliaceae

**DOI:** 10.1186/s12870-020-2326-5

**Published:** 2020-03-12

**Authors:** Fabian Bratzel, Sascha Heller, Nadine Cyrannek, Juraj Paule, Elton M. C. Leme, Anna Loreth, Annika Nowotny, Markus Kiefer, Walter Till, Michael H. J. Barfuss, Christian Lexer, Marcus A. Koch, Georg Zizka

**Affiliations:** 1 Department of Botany and Molecular Evolution, Senckenberg Research Institute and Natural History Museum Frankfurt, Senckenberganlage 25, 60325 Frankfurt am Main, Germany; 2 Centre for Organismal Studies (COS) Heidelberg, Department for Biodiversity and Plant Systematics, University of Heidelberg, Im Neuenheimer Feld 345, 69120 Heidelberg, Germany; 3 Institute for Ecology, Evolution and Diversity, Goethe University, Max-von-Laue-Straße 13, 60438 Frankfurt am Main, Germany; 4 Marie Selby Botanical Gardens, 811 South Palm Avenue, Sarasota, FL 34236 USA; 5 Department of Botany and Biodiversity Research, Faculty of Life Sciences, University of Vienna, Rennweg 14, 1030 Vienna, Austria

**Keywords:** DNA barcoding, Low-copy nuclear gene, Plant collections, Bromeliads

## Abstract

**Background:**

The angiosperm family Bromeliaceae comprises over 3.500 species characterized by exceptionally high morphological and ecological diversity, but a very low genetic variation. In many genera, plants are vegetatively very similar which makes determination of non flowering bromeliads difficult. This is particularly problematic with living collections where plants are often cultivated over decades without flowering. DNA barcoding is therefore a very promising approach to provide reliable and convenient assistance in species determination. However, the observed low genetic variation of canonical barcoding markers in bromeliads causes problems.

**Result:**

In this study the low-copy nuclear gene *Agt1* is identified as a novel DNA barcoding marker suitable for molecular identification of closely related bromeliad species. Combining a comparatively slowly evolving exon sequence with an adjacent, genetically highly variable intron, correctly matching MegaBLAST based species identification rate was found to be approximately double the highest rate yet reported for bromeliads using other barcode markers.

**Conclusion:**

In the present work, we characterize *Agt1* as a novel plant DNA barcoding marker to be used for barcoding of bromeliads, a plant group with low genetic variation. Moreover, we provide a comprehensive marker sequence dataset for further use in the bromeliad research community.

## Background

The rapidly radiating monocot plant family Bromeliaceae is currently considered to comprise 3597 species and 76 genera [1] with a geographical distribution mainly confined to the Neotropics [2, 3]. Bromeliads are a morphologically highly distinctive plant group, for which an up-to-date monograph is missing for most of the genera. Thus, determination requires great expertise up to the point where some species of problematic groups might only be confidently identified by few specialists. Moreover, determination often relies on generative characters, while flowering occurs rarely in many bromeliads, especially when cultivated in Botanical Gardens in the northern hemisphere. Thus, methods for assistance in determination, such as DNA barcoding, are of high interest for the bromeliad research community. This particularly applies to the Botanic Garden context, as bromeliads often comprise a substantial portion of the collections, due to their popularity as ornamental plants, as well as their scientific value, e.g. as a model group for rapid radiations and evolution of CAM photosynthesis [3–5].

Within the scope of an initiative to improve access to and usability of living plant collections of Cactaceae and Bromeliaceae in Botanical Gardens in Germany (EvoBoGa) [6], we aim to develop an easy to handle and cost effective DNA barcoding protocol as well as to provide a comprehensive barcode database for bromeliads.

The DNA barcoding approach is widely used to identify animal species and within the scope of the International Barcode of Life (IBOL) project, a great number of animal species can be successfully identified using genetic markers [7]. For animal barcoding, the cytochrome c oxidase I (COI) locus of the mitochondrial genome is universally used while in plants the same locus is not informative, thus alternatives are required [8, 9]. Plastid markers such as *matK* and *rbcL* as well as nuclear markers such as ITS1/ITS2 and their combinations have been suggested for plant barcoding, but the applicability is not universal thus different groups require different barcoding markers [10–13].

In recent years, some efforts have been made to test a set of canonical plant barcoding markers in Bromeliaceae. However, it was demonstrated that *rbcL*, *trnH-psbA* and *matK* are not sufficient for species determination, due to the low genetic variation [14]. Thus, developing a bromeliad specific barcoding procedure requires the assessment of new potential DNA barcoding markers and eventually the consideration of new approaches to barcoding.

In the present study, we examine the potential of the low-copy nuclear gene (LCNG) *Agt1* as a marker for Bromeliaceae DNA barcoding. *Agt1* encodes a glyoxylate aminotransferase that is involved in the photorespiration pathway in *Arabidopsis* [15–17] and the locus was suggested as a marker for phylogenetic studies at low taxonomic levels [18]. Since then *Agt1* has been successfully used in a number of phylogenetic studies covering a wide range of angiosperm plant groups [19–25]. In two recent studies that aimed at a revision of the “Cryptanthoid complex” [23] and the genus *Ananas*, including its closest relatives [25], *Agt1* proved to be of great value for the reconstruction of Bromeliaceae genus/subgenus level phylogenies and thus prompted a further investigation of its potential suitability for barcoding.

Based on these objectives, we selected well-determined and phylogenetically classified representatives from major bromeliad subfamilies and generated *Agt1* sequences in order to: (1) examine the genetic diversity and evaluate the intraspecific variation for presence of a “barcoding-gap”; (2) elucidate the phylogenetic relevance and limitations of the *Agt1* sequence information; (3) assess the MegaBLAST based species determination rate and compare it with *matK*; and (4) evaluate the resolution below species level for population-studies and for potential assistance of *Agt1* in tracing putative hybrid origins of bromeliad species.

## Results

### *Agt1* PCR amplification and sequencing success rates

The *Agt1* marker region used for this study was initially amplified as described in previous studies [18, 23]. As double bands occurred at high frequency, we designed bromeliad specific primers with higher annealing temperatures, to attain increased specificity and to impede the occurrence of unspecific bands. Hereby, we were able to obtain the 300–700 base-pairs (bp) long *Agt1* PCR fragments in 370 of 415 attempted cases (89.2%) where sufficient DNA quality was verified through gel electrophoresis and fluorometric quantitation.

Moreover, Sanger sequencing success rates were rather low when using the *Agt1* specific primers also for sequencing. Hence, we added universal sequencing primer sites (M13, SP6) to each oligonucleotide. Considering only those cases as a success in which sequencing worked for both primers and thus resulted in a full-length consensus sequence alignment, the *Agt1* sequencing success rate was at 287 out of 370 (77.5%). Taken together, we obtained 579 *Agt1* Bromeliaceae barcodes for this study, covering 236 Tillandsioideae, 3 Hechtioideae, 3 Navioideae, 38 Pitcairnioideae, 10 Puyoideae, 1 Brocchinioideae and 288 Bromelioideae taxa, some of which were already published in earlier studies [23, 25].

In 51 cases (approximately 15%) we were not able to successfully sequence the *Agt1* intron IV portion as the electropherograms displayed double peaks, probably due to allelic variation within the intron. In order to investigate this in more detail we also cloned *Agt1* alleles of selected accessions and found allelic differences occurring due to insertions/deletions within the intron IV in all examined cases (Additional file 4: S4). These allelic differences were confined to the intron IV, within the exon IV sequence portion we could not detect a significant sequence variation.

### *Agt1* sequence characteristics and sequence clustering

**Fig. 1:**
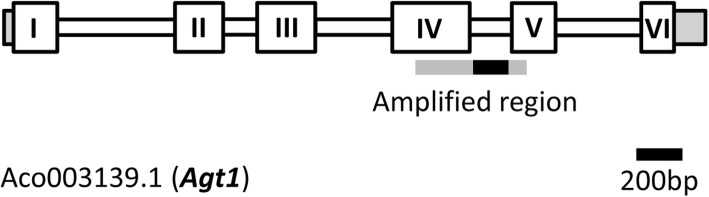
Gene model of the *Ananas comosus Agt1*-locus (Aco003139.1) modified from *Phytozome* [26, 27]. Grey boxes are untranslated regions. Numbered Boxes are exons, unnumbered boxes depict introns. Shown in grey/black: *Agt1* exon IV/intron IV region amplified in this study

The portion of the *Agt1* gene body amplified comprises approximately three-quarters of exon IV (264 bp), the entire intron IV, as well as a very short portion of exon V (14 bp), as depicted in Fig. 1. In contrast to the *Agt1* ortholog from *Arabidopsis thaliana*, which is 1486 bp in size, the bromeliad gene body is about 3025 bp long and contains five instead of four exons (Additional file 7: S7). The Bromeliaceae *Agt1* intron IV region proved to be highly variable among the *Agt1* sequences in our database, ranging from approximately 100 base pairs (bp) to about 400 bp. When trying to align intron IV of all accessions, we found presence of insertions/deletions at a high frequency, not only among distantly related taxa, but also within species of the same subfamilies. Thus, we were not able to generate an alignment over the whole Bromeliaceae family neither over subfamilies.

As in previous studies it was shown that sequence divergence within the *Agt1* intron IV on the genera/clade level is rather low and thus aligns well [23, 25], we assessed the *Agt1* sequence similarity among the clades reported in these two studies and found that values are at ≥97% sequence identity. Given this, we aimed to find clusters of sequence identity above 98% among all *Agt1* sequences present in our database, using the DNA sequence clustering software CD-HIT-EST [28].

As shown in Table 1, we found 52 clusters with a sequence identity ≥98% among the *Agt1* sequences from the subfamily Bromelioideae, 34 clusters among the Tillandsioideae and 8 clusters among the Pitcairnioideae.

**Table 1:** Clusters of sequence identity ≥98% among *Agt1* sequences from three Bromeliaceae subfamilies using the DNA sequence clustering software CD-HIT-EST [28]

Subfamily	Number of sequences	Number of species	Number of CD-HIT-EST Clusters	Clusters with/without support	Species clusters
Bromelioideae	**288**	**231**	**52**	**31/9**	**12**
Tillandsioideae	**203**	**155**	**34**	**21/10**	**3**
Pitcairnioideae	**41**	**39**	**8**	**8/0**	**0**

### Phylogenetic validity of *Agt1* sequence clusters

Support for phylogenetic relevance of the *Agt1* sequence-clustering in Bromelioideae was obtained from a recent work about the "*Portea*/*Gravisia* complex [29]. The corresponding sequences present in our *Agt1* dataset cluster with identity values above 98% and no non-“*Portea*/*Gravisia* complex” members are present within this cluster. Another six clusters represent groups recently assigned to the “Cryptanthoid complex” [23], five are members of the “Nidularioid complex” [30, 31] and one comprises only taxa of the recently studied genus *Ananas* [25]. Among the remaining Bromelioideae *Agt1* sequences we found seven clusters that consist only of members from either one of the following genera: *Wittmackia*, *Billbergia*, *Araeococcus*, *Hohenbergia*, *Lymania* or *Neoregelia*. All of these genera are currently considered as being monophyletic [29–38].

While for 31 of the sequence clusters we got phylogenetic support, 9 of the detected Bromelioideae clusters exhibit a species composition that is not in accordance with current classifications. Twelve of the Bromelioideae *Agt1* clusters are comprised of members from the same species.

In case of the 34 *Agt1* sequence clusters we detected among Tillandsioideae members, we refer to two recently published studies, based on multi-locus DNA sequence and morphological data [1, 39, 40]. As shown in Table 1, we found 21 clusters with literature support and 10 clusters that contain taxa from different clades and are thus contradicting to the literature (Additional file 2: Table S2). Moreover, within all clusters we found taxa that have not yet been included in any phylogenetic study.

The *Agt1* sequences we obtained from Pitcairnioideae members all clustered with members of the same genus [1, 41]. Accordingly, we detected five clusters containing only species from either the monophyletic genus *Pitcairnia*, *Fosterella* and *Deuterocohnia*, respectively (Additional file 2: Table S2).

### Genus level genetic diversity and “barcoding-gap” assessment

**Fig. 2:**
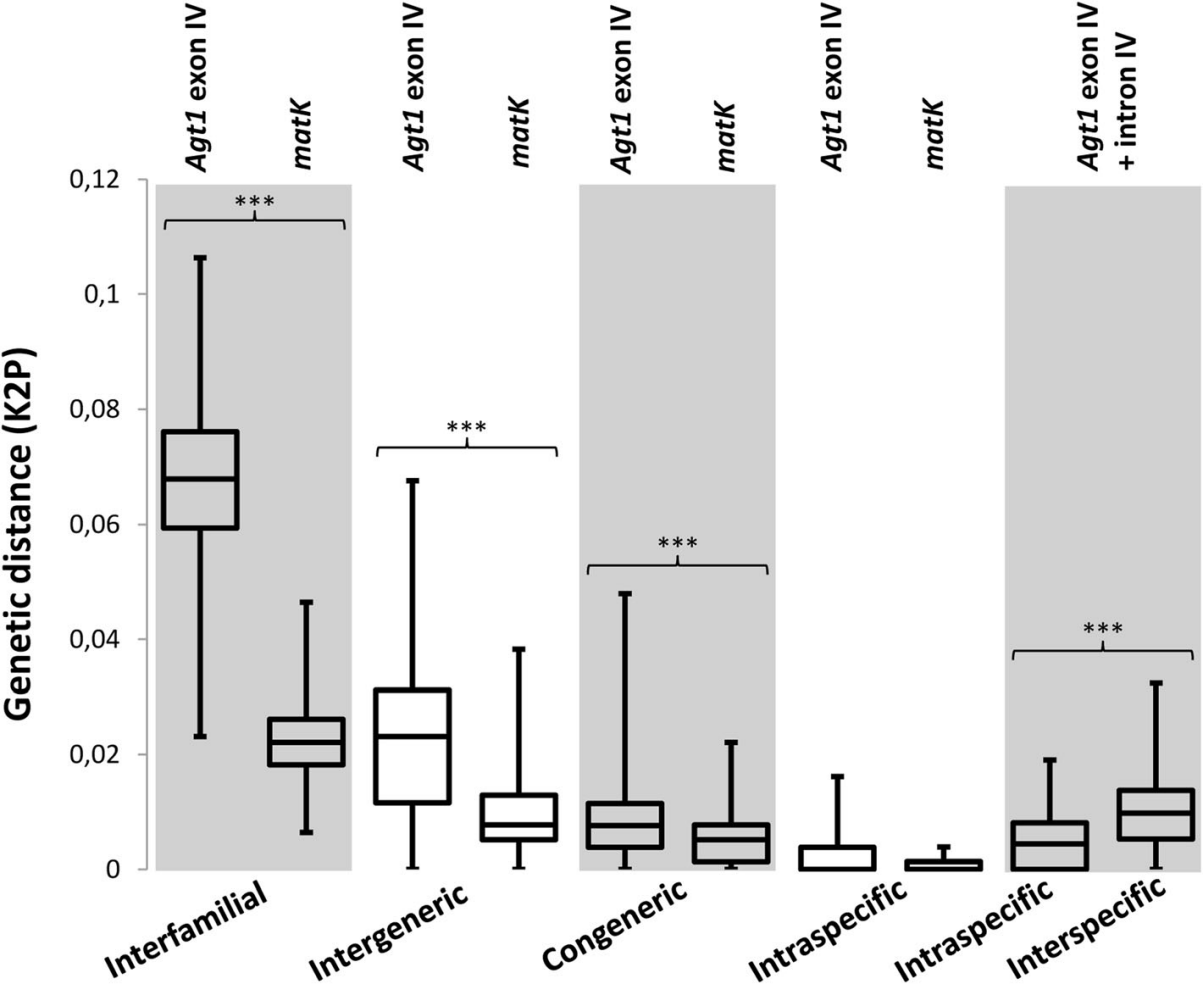
Genetic divergence (K2P) among *Agt1* exon IV (*n* = 234), *matK* Sequences (*n* = 233) among different taxonomic levels, as well as *Agt1* exon IV (*n* = 45, Tillandsioideae) and *matK* (*n* = 58, Tillandsioideae) from different accessions of Tillandsioideae species (Intraspecific). Right-most pane: Genetic Divergence between *Agt1* exon IV + intron IV for a subset of Tillandsioideae species to examine presence of a “barcoding-gap” (*n* = 97 Interspecific and *n* = 53 Intraspecific). Asterisks indicate statistical significance based on the Wilcoxon rank sum test (*** *p* < 0.001)

To assess the genetic diversity of the *Agt1* exon IV/intron IV sequences, we generated alignments of the clusters with a sequence similarity above 98% and calculated the genetic diversity using the Kimura 2-parameter (K2P) model (Additional file 5: S5). As shown in Fig. 2 (right-most pane), the K2P values among different species (interspecific) range between 0,005 and 0,015 with outliers and a median at approximately 0.01.

In order to assess whether we find differences between the K2P values among species and within a given species (intraspecific), we also calculated the genetic diversity among accessions of the same species. Due to practical reasons, this analysis was confined to 11 species from the subfamily Tillandsioideae, with an average of 5 accessions per species. As shown in Fig. 2 (right-most pane), the K2P values overlap and thus we can not find a “barcoding-gap”.

### Family level genetic diversity and comparison with *matK*

Due to the high genetic variabilities of the *Agt1* intron IV portion and the resulting difficulties with generating alignments, we split the exon IV/intron IV sequences and removed the 14 bp portion from exon V for further analysis. The exon/intron boundary was determined according to the annotated *Ananas comosus Agt1* gene model and peptide sequence [26].

Evaluation of the obtained sequence data demonstrated that the *Agt1* exon IV region has a portion of 31% variable sites and the rate of parsimony-informative sites is at about 23% (Table 2 and Additional file 5: S5). For this assessment we used the K80 substitution model as it was reported to be most accurate for the *Agt1* marker in bromeliads [23].

**Table 2:** *Agt1* and *matK* marker region properties. Alignment length, phylogenetic information content and substitution models used

Marker region	Alignment length (bp)	Variable sites	Parsimony-informative sites	Substitution model
*Agt1* exon IV	**264**	**82/264 (31%)**	**60/264 (22,7%)**	**K80**
*matK*	**782**	**189/782 (24,2%)**	**113/782 (14,5%)**	**K80**

As *matK* was reported to be the most promising Bromeliaceae DNA barcoding marker [14], we also included *matK* in this study. Therefore, we downloaded 440 *matK* sequences covering a wide portion of the Bromeliaceae genera used for this study from GenBank and evaluated the genetic variation exactly as we did with *Agt1* (Additional file 6: S6). As shown in Table 2, the rate of variable sites (24%) as well as the rate of parsimony-informative sites (14,5%) of *matK* is considerably lower as in the case of *Agt1*.

We next compared the genetic diversity of the *Agt1* and *matK* sequences based on the K2P substitution model. The *Agt1* exon IV sequence alignments were compared between taxa from different subfamilies (Interfamilial), between species groups within the subfamilies (Intergeneric), among species from species groups within each subfamily (Congeneric). As shown in Fig. 2, compared to *matK*, *Agt1* shows relatively high genetic distance values among all taxonomic levels. The mean interspecific distances of *Agt1* are considerably higher than the intraspecific distances however, the values are overlapping, indicating that in a number of cases, the sequence divergence of the *Agt1* marker region within species is not lower than among species.

### *Agt1* mediated species identification using MegaBLAST

In order to assess the applicability of *Agt1* as a DNA barcoding marker and to compare its performance with that of *matK*, we generated a sequence-database that contained all exon IV/intron IV *Agt1* sequences. To perform a MegaBLAST search against this Bromeliaceae *Agt1* database, we used all those taxa of which we had sequences from more than one different provenance. Hereby, we were able to search 41/42 species as queries against the database that was comprised of 567 different species, covering the Bromeliaceae subfamilies Brocchinioideae, Bromelioideae, Hechtioideae, Navioideae, Pitcairnioideae, Puyoideae and Tillandsioideae. As shown in Table 3 and Additional file 2: Table S2, correct species identification using *Agt1* exon IV/intron IV was possible in 22 of 41 tested cases.

**Table 3:** Identification-success rates of a local MegaBLAST search against the Bromeliaceae *Agt1* and *matK* databases

Marker	Taxa level	Number of species tested	Ambiguous	Correctly identified
*Agt1* (exon IV)	**Species**	**42**	**3**	**10**
*Agt1* (exon IV + intron IV)	**Species**	**41**	**4**	**22**
*Agt1* (exon IV + intron IV)	**Genus/Clade**	**42**	**–**	**32**
*matK*	**Species**	**42**	**2**	**10**

We also tested species identification rates for *matK* using also 42 species as queries against a *matK* database containing 440 sequences in total. As indicated in Table 3 and Additional file 2: Table S2, the identification success corresponded to 10 out of 42 and is thus significantly lower as in the case of *Agt1*.

We next tested the correct assignment to species groups as they were defined for the subfamilies Bromelioideae and Tillandsioideae [23, 39]. In 32 out of 42 tested cases, the *Agt1* sequence with the highest Bit-score was assigned to a species that is considered in the same species-complex or species group (Table 3 and Additional file 2: Table S2).

### Application of DNA barcoding beyond species identification

As preliminary studies indicated that *Agt1* sequence diversity might be also applicable to resolve population level diversity and to eventually assists in investigating hybridization [23, 25], we obtained *Agt1* sequences from putative hybrid species as well as from their potential parents.

**Fig. 3:**
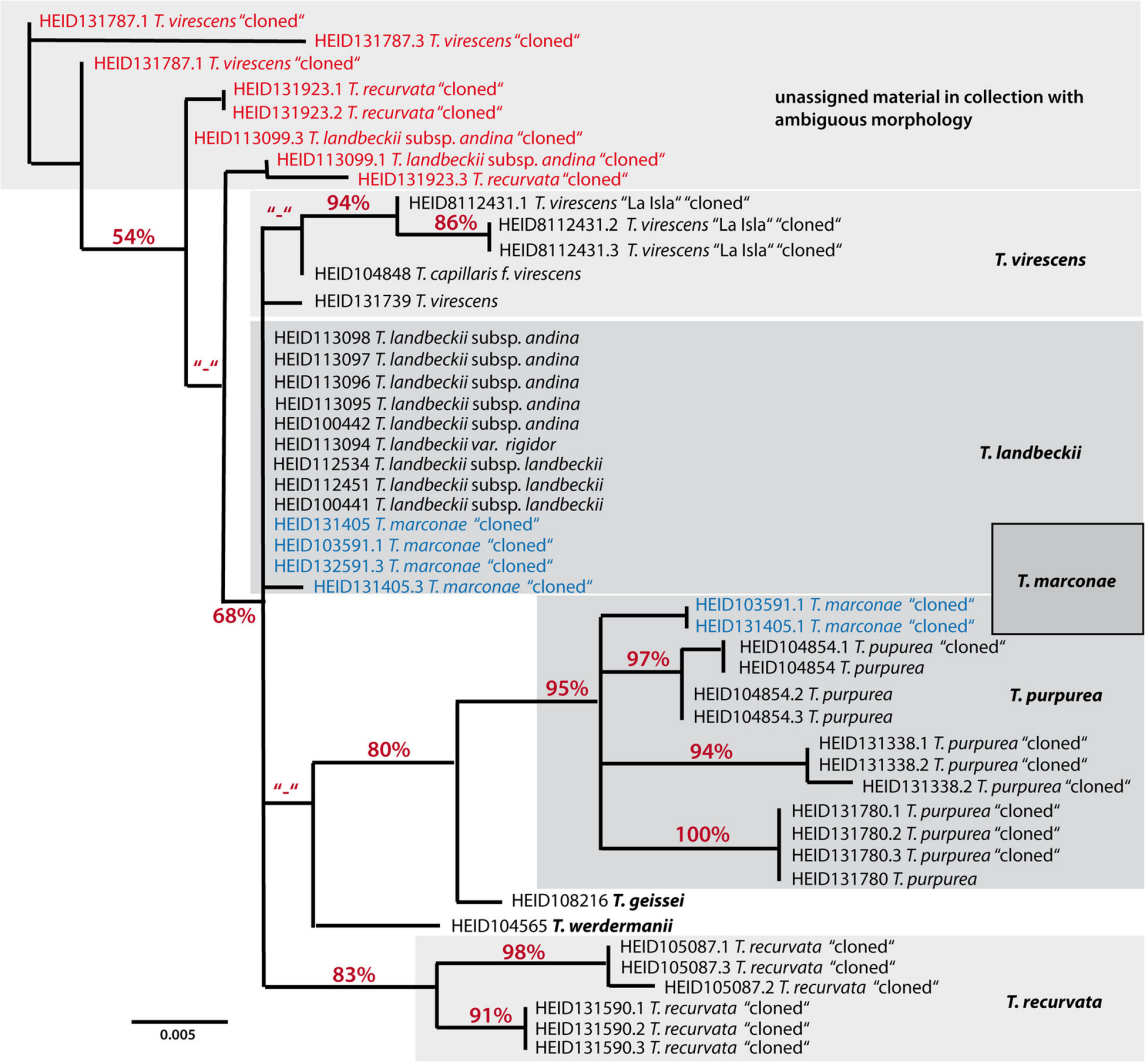
*Agt1* exon IV + intron IV maximum likelihood tree of various accession from the *Tillandsia* subgenus *Diaphoranthema* members *T. virescens*, *T. recurvata* and *T. landbeckii* as well as *Tillandsia purpurea* complex members (including *T. marconae*). “cloned” indicates that the *Agt1* sequences were cloned to test for *Agt1* copy number and paralog variations. Numbers correspond to accession codes from Heidelberg Botanical Garden (accession details are found with Table S1). Allopolyploid hybrid *T. marconae* is indicated in blue font. Accessions in red font indicate ambiguous morphological characters and might represent additional accessions of hybrid origin

We chose the *Tillandsia* subg. *Diaphoranthema* member *Tillandsia marconae* for this purpose, as it has been described as a putative hybrid species of *Tillandsia paleacea* and *Tillandsia purpurea* [42]. First, we cloned the *Agt1* sequences from the potential parent species accessions *Tillandsia purpurea*, *T. virescens*, *T. recurvata* and *T. landbeckii*, that were collected from sites of co-occurrence in southern Peruvian deserts. As the *Agt1* maximum likelihood tree shown in Fig. 3 depicts, *T. virescens*, *T. recurvata* and *T. landbeckii* that have been assigned to the *Tillandsia* subg. *Diaphoranthema* in previous studies [39, 40, 43], can be clearly separated from the *Tillandsia purpurea* complex members *T. purpurea* and *T. marconae*. The different *T. marconae Agt1* alleles are clearly assigned either to the *T. landbeckii* or the *T. purpurea* genepool (identical alleles and/or high bootstrap support). *Tillandsia marconae*, thereby, carries alleles from the genepools of both parental species, which coincides also with a local endemic occurrence in northern Chile mediating between Peruvian *T. purpurea* and largely Chilean *T. landbeckii*. Accordingly, we interpret *T*. *marconae* as a putative hybrid with *T. purpurea* and *T. landbeckii* from subg. *Diaphoranthema* as parents as suggested earlier [42, 43].

## Discussion

### Genetic diversity and phylogenetic validity of *Agt1* sequences

We obtained 579 *Agt1* exon IV + intron IV sequences covering a wide range of the Bromeliaceae subfamilies Bromelioideae, Tillandsioideae, Pitcairnioideae, Puyoideae, Brocchinioideae and Hechtioideae. Due to the high variation of the *Agt1* intron IV sequence portion and since only for a subset of included species phylogenetic information is available, we could not generate a global and family-wide alignment. Instead, we performed a sequence cluster analysis using the software CD-HIT-EST, to obtain groups with sequence identity values ≥98%. We chose the threshold at 98% because the *Agt1* sequence similarity among the clades reported in previous studies, range at about 98% and above [23, 25]. When comparing these clusters with published phylogenies, we found that in the majority of cases, the clusters represented currently accepted taxonomic groups (Table 1). These groups either consisted of individuals of the same species, of species from the same genus or of species that where assigned to a subgenus or a species group in the literature.

Consequently, the genetic diversity of the *Agt1* sequences corresponds fairly well to currently accepted multi-locus phylogenies and seems to be well suited to be used as a DNA barcoding marker throughout the Bromeliaceae. However, as some of the obtained clusters contained species not fitting to the respective group and additionally, as within non of the clusters we found all of the species we expected to find - although present within the database - it is obvious that use of *Agt1* as a DNA barcoding marker requires precaution, as not for all species safe determination will be possible.

Having clearly separated magnitudes between the intraspecific and interspecific genetic variation (“barcoding-gap”) is considered a pivotal requirement for a good DNA barcoding marker [44], although the issue is controversially discussed [45]. We assessed the differences among the *Agt1* exon IV as well as *Agt1* exon IV + intron IV Kimura 2-parameter (K2P) values between species and among different accession. As shown in Fig. 2 (right-most pane), we found overlap between the intra- and interspecific K2P values within the tested cases and thus we can not discern a distinct “barcoding-gap”. However, as it is questionable whether occurrence of such a gap can be expected to be realistic at all or whether it might only be an artefact due to insufficient sampling [46], we do not consider lack of a “barcoding-gap” a general objection against use of *Agt1* for DNA barcoding.

Principally, we consider that due to gene-flow and depending on the evolutionary divergence of closely related species, we will have to deal with the problem of insufficient *Agt1* sequence divergence, and in certain cases DNA barcoding will generally be challenging. This circumstance was shown for example in our MegaBLAST based species identification trial, where we found ambiguities in 4 out of 41 tested cases (Table 3). In these instances we found one or many other species displaying the same Bit-Score as the query species. Given the relatively limited species number of these trials, we additionally have to assume that the more species will be added to the database in the future, the more we will have to deal with such ambiguities due to limited *Agt1* sequence divergence.

### Species discrimination using *Agt1* versus *matK*

Performance of a MegaBLAST search of selected taxa against our bromeliad *Agt1* sequence database allowed us to correctly identify 22 of the 41 tested species (53%). Accordingly, using *Agt1* we can increase the discriminatory success of DNA barcoding for Bromeliaceae with a single marker region about two fold, compared to a previous study which reported 27.6% identification success for *matK*, 19% for *rbcL* and 26% for *trnH-psbA* [14]. The same authors reported a maximum of 44.4% identification success when the three markers *rbcL* + *matK* + *trnH-psbA* were combined, which is still considerably lower than *Agt1* [14].

An identification success rate of about 53% appears generally rather low when compared to studies that aimed to discriminate among heterogeneous and not closely related groups, such as medical plants or species of a rainforest patch, which successfully identified up to 90% of the species [10, 47]. However, a number of recently published studies demonstrate that DNA barcoding of closely related species is in all reported studies much less efficient than within more heterogeneous groups. For instance, using ITS2 allowed to correctly identify 76.4% of investigated Asteraceae species [48]. In a study that aimed to test ITS for usage as a barcoding marker within the genus *Corydalis* (Papaveraceae) an identification success rate of 65.2% was reported [49]. Two other studies investigated the use of the nuclear marker ITS2 for barcoding of closely related *Curcuma* species (Zingiberaceae) and found that 46.7% respectively 73% of species were successfully identified [50, 51].

Accordingly, our correct species identification rate of about 53% is within the range reported for other Angiosperm groups and we conclude that due to the above discussed reasons it might be generally difficult to obtain higher scores, especially in groups with a well known low genetic variation such as the bromeliads [14].

### Applicability of DNA barcoding in Bromaliaceae using *Agt1*

Given a PCR success rate of 89%, a sequencing success rate of 77% and taking into account the fact that we were able to recover full-length *Agt1* sequences from seven of eight Bromeliaceae subfamilies, we consider the barcode universally utilizable throughout the family. In many other studies the reported amplification efficiencies for markers such as *matK* and ITS1/2 are within the same range or even lower [52, 53].

The fact that allelic variation of the intron IV portion detained sequencing and required cloning in about 15% of the cases is partially contradicting our prerequisite of finding a marker that allows us to develop a cheap and easy to handle barcoding procedure. However, it also bears the chance to expand its applicability to be extended to study provenances of accessions in plant collections as well as to assist in population studies or detecting potential F1 or early generation hybrids.

In order to test the possibility that the high genetic variability of *Agt1* might suffice to investigate the reticulate evolution of bromeliad species, we conducted a case-study using the *Tillandsia* subg. *Diaphoranthema* member *Tillandsia marconae*, as it has been described as a potential hybrid species of *Tillandsia paleacea* and *Tillandsia purpurea* [42]. From our results (Fig. 3) we have good indications that *Tillandsia marconae* might be a hybrid of *Tillandsia purpurea* and another subg. *Diaphoranthema* member, as one allele of both cloned species is closest to the allelic genepool of respective parental species in our maximum likelihood analysis (Fig. 3). Given the fact that among the included taxa only *Tillandsia landbeckii* shares the habitat with *Tillandsia marconae* and *Tillandsia purpurea* [54], we consider it likely that *Tillandsia marconae* arose through hybridization of *Tillandsia landbeckii* and *Tillandsia purpurea*. These findings are in support of an earlier study that used other low-copy nuclear markers to address the same question [43]. Although these hypothesis needs to be further underlined with other taxonomically relevant data and with a more comprehensive dataset, we consider it noteworthy to find that the *Agt1* sequence resolution is high enough to be also used for studies on the population level in order to reconstruct reticulate evolutionary processes.

## Conclusion

Taken together, we demonstrate that *Agt1* allows to score correct Bromeliaceae species determination rates of about 53% (22 out of 41 cases), which corresponds to a two-fold increase of previously reported rates using single markers which were at maximum 27.6% and at 44.4% for an elaborate combination of three markers [14]. The correct species identification rate might be further increased by additionally using other canonical markers and even more by extending the dataset to genome scale in the future.

As we intend to develop an easy to handle protocol that can be applied in the Botanical Garden context with low cost effort and basic supplied lab-facilities, we consider using *Agt1* a possible solution for assistance in bromeliad species determination and potentially also other plant groups with low genetic variation. The respective knowledge database system referring to documented reference material is under development.

## Methods

### Plant material acquisition and sampling strategy

Plant material was collected from three botanical gardens in Germany and Austria (Botanischer Garten Heidelberg, Germany; Alter Botanischer Garten Göttingen, Germany; Botanischer Garten und Botanisches Museum Berlin, Germany; Botanischer Garten der Universität Wien, Austria) as well as from a private collection of one of the co-authors (EMCL) in Brazil (Refúgio dos Gravatás, Teresópolis, Rio de Janeiro, Brazil). The living specimens were grown following the guidelines recommended by article 9 of the Convention on Biological Diversity for ex situ conservation (1993). Our criterion for sufficient taxonomic classification of the sampled material was a verifiable evaluation by at least one renowned Bromeliaceae expert and full documentation of species collection history (e.g. “The Werner Rauh Heritage Project” [55]). Provenance of the plant material, names of the persons that identified the plant material and information about availability of voucher specimens are listed in Additional file 1: Table S1.

We attempted to cover all clades that were represented in a recent revision of the Tillandsioideae subfamily [39], as well as all clades recovered in recent studies of Bromelioideae [23, 25, 32, 34–36, 56]. Of the subfamilies Brocchinioideae, Hechtioideae, Navioideae, Pitcairnioideae and Puyoideae we added as many different species as we could obtain from any of the collections mentioned above. This finally resulted in 477 species (of 3597) and 51 out of currently accepted 76 genera [1].

We additionally obtained plant material from the presumed hybrid species *Tillandsia marconae* W. Till & Vitek as well as a number of potential parental species (*T. paleacea* C. Presl, *T. purpurea* Ruiz & Pav., *T. virescens* (Ruiz & Pav.) L. B. Sm., *T. recurvata* (L.) L. and *T. landbeckii* Phil.) collected from sites in southern Peruvian deserts [42].

### DNA extraction, PCR amplification, DNA sequencing and plasmid cloning

DNA extraction from fresh or dried leaf material was carried out using the Qiagen (Venlo, NL) DNeasy Plant Mini Kit, according to the manufacturers instructions. PCR amplification of the *Agt1* marker region was performed using MyTaq DNA-polymerase (Bioline, London, UK) using 10–20 ng DNA template, 5x MyTaq reaction buffer (5 mM dNTPs and 15 mM MgCl), 10 pmol of each primer (AGT1_SP6_Fw: 5‘-ATTTAGGTGACACTATAGATTGATGTCGCATTAACCGGC-3‘ and AGT1_M13_Rev: 5’-AACAGCTATGACCATGGCAGTTCTTCAGTCCCCATG-3’). The cycling conditions were the following: 95 °C 3 min. [30 cycles: 95 °C 30 s.; 56 °C 20 s.; 72 °C 20 s.] 72 °C 5 min. Before designing bromeliad specific primers, we also used the canonical *Agt1* primers that were used in previous studies [18, 23]. PCR amplification and sequencing of *matK* was performed using the primer combination MatK 5F: 5′-ATACCCTGTTCTGACCATATTG-3′ and trnK2r 5′-AACTAGTCGGATGGAGTAG-3′. Internal sequencing of *matK* was done using the primer TOmatK 480F 5′-CATCTKGAAATCTTGGTTC-3′ [57].

Prior to sequencing, PCR reactions were cleaned-up by combined treatment with Exonuclease I (New England Biolabs, Ipswich, MA, USA) and Shrimp Alkaline Phosphatase (Thermo Fischer Scientific, Waltham, MA, USA) according to the supplier recommendations.

Sanger Sequencing was performed using an ABI 3730 platform (Applied Biosystems, Foster City, CA, USA) device at the BiK-F Sequencing core facility (Senckenberg Biodiversity and Climate research center, Frankfurt, Germany) using standard sequencing primers (SP6_Fw 5′-ATTTAGGTGACACTATAG-3′ and M13_Rev 5′-AACAGCTATGACCATG-3′).

*Agt1* PCR fragment cloning was done using the CloneJET PCR Cloning Kit (Thermo Fischer Scientific, Waltham, MA, USA) according to the supplier recommendations. Four to five plasmids per cloning assay were then sequenced at Eurofins Genomics (Eurofins Scientific, Luxemburg, LU) using universal plasmid sequencing primers M13 forward and SP6 reverse.

### Acquisition of *matK* sequences from GenBank

482 *matK* sequences were downloaded from GenBank. All sequences were aligned and trimmed to a length of 782 bp. A list with the GenBank accession numbers of all species included can be found in Additional file 1: Table S1.

### Data analysis

Editing and analysis of the sequence data were done using the *Geneious* software (Biomatters, Auckland, NZ, Version 11.0.5) [58]. Sequence alignments were carried out using the *Geneious* implemented *MAFFT* sequence alignment tool (Version 7.388) [59]. Sequence alignments were further analyzed using the *MEGA7* software package [60]. Kimura 2-parameter Distance (K2P) analysis of the exon IV sequence alignments was also done using *MEGA7*, statistical significance was tested using the Wilcoxon rank sum test.

*Agt1* sequence database cluster analysis was performed using the software CD-HIT-EST suite [28, 61]. The final sequence identity cut-off was set at 0.98, all other parameters were set at default (Additional file 2: Table S2).

BLAST mediated species identification was performed using the *Geneious* implemented custom BLAST function [59]. All FASTA sequences of the *Agt1* exon IV were trimmed to a length of 264 bp and the intron IV portions were cut-off at the exon V boundary. Database search was done by pairwise comparison using the MegaBLAST algorithm with the use of the following settings: Scoring (Match Mismatch): 1–2; Gap cost (Open Extend): linear; Max E-value: 10; Word Size: 28. The output was ordered by increasing Bit-Score for each hit. We considered identification to be successful only in those cases where the highest Bit-Score corresponded to the same species as the Query and where all other species did have a disparate lower Bit-Score (Additional file 2: Table S2).

The maximum likelihood tree was generated using PAUP* [62]. Total length of the alignments was 492 bp, “Gaps” and missing data were not counted (Fig. 3 and Additional file 3: S3). Bootstrap support values (> 50%) from 1000 replicates are provided.

## Supplementary information

**Additional file 1.** Supplementary_Table_S1_Plant_Material.

**Additional file 2.** Supplementary_Table_S2_CD_Hit_MegaBLAST.

**Additional file 3.** Supplementary_Material_S3_Agt1_Alignment_Tillandsia_ML_Tree.

**Additional file 4.** Supplementary_Material_S4_Agt1_Cloning.

**Additional file 5.** Supplementary_Material_S5_Agt1_exonIV_alignment.

**Additional file 6.** Supplementary_Material_S6_matK_alignment.

**Additional file 7.** Supplementary_Material_S7_Agt1_genmodels_Acomosus_Athaliana.

## Data Availability

The datasets used for this study are included in the manuscript and the Supplementary Materials. All DNA sequences obtained for this study were submitted to GenBank, the accession numbers are listed in Additional file 1: Table S1.

## Abbreviations

bp: base-pairs
IBOL: International Barcode of Life
K2P: Kimura 2-parameter
LCNG: low-copy nuclear gene
PCR: Polymerase Chain Reaction
subg.: Subgenus
