# Correction to: The low-copy nuclear gene *Agt1* as a novel DNA barcoding marker for Bromeliaceae

**DOI:** 10.1186/s12870-020-02743-3

**Published:** 2020-12-30

**Authors:** Fabian Bratzel, Sascha Heller, Nadine Cyrannek, Juraj Paule, Elton M. C. Leme, Anna Loreth, Annika Nowotny, Markus Kiefer, Walter Till, Michael H. J. Barfuss, Christian Lexer, Marcus A. Koch, Georg Zizka

**Affiliations:** 1 Department of Botany and Molecular Evolution, Senckenberg Research Institute and Natural History Museum Frankfurt, Senckenberganlage 25, 60325 Frankfurt am Main, Germany; 2 Centre for Organismal Studies (COS) Heidelberg, Department for Biodiversity and Plant Systematics, University of Heidelberg, Im Neuenheimer Feld 345, 69120 Heidelberg, Germany; 3 Institute for Ecology, Evolution and Diversity, Goethe University, Max-von-Laue-Straße 13, 60438 Frankfurt am Main, Germany; 4 Marie Selby Botanical Gardens, 811 South Palm Avenue, Sarasota, FL 34236 USA; 5 Department of Botany and Biodiversity Research, Faculty of Life Sciences, University of Vienna, Rennweg 14, 1030 Vienna, Austria

**Correction to: BMC Plant Biol 20, 111 (2020)**

**https://doi.org/10.1186/s12870-020-2326-5**

In the original publication [1] an incorrect version of Additional file 1 was used during typesetting. The incorrect and correct versions of Additional file 1 are available in this correction article. The original article has been updated. The publisher apologizes to the authors and readers for the inconvenience.

## Supplementary Information

**Additional file 1:** Incorrect version of Additional file 1.

**Additional file 2:** Correct version of Additional file 1.
