# Supplementary material for: The low-copy nuclear gene Agt1 as a novel DNA barcoding marker for Bromeliaceae
Source: BMC Plant Biol. 2020 Mar 12;20:111. doi: 10.1186/s12870-020-2326-5 (PMC7068990; doi:10.1186/s12870-020-2326-5)
Supplement: Supplementary file 7 — Additional file 7. Supplementary_Material_S7_Agt1_genmodels_Acomosus_Athaliana. [file 12870_2020_2326_MOESM7_ESM.pdf]

5'-UTR

CDS

3'-UTR

Arabidopsis thaliana Agt1* (AT2G13360.1) *genemodel extracted from PHYTOZOME v12.1

gcaagctacttgattgggataaactaaataacaaaataaactacttacaaaaacatcatgtaatttaaaataaataaataaaataaaaaaaaga
aaaagaagaagatatattaaagaggtttgtgaagacgaaggagtccaaaacctccctcaggagcttccgccttattcagaggttaaacttctctc
ttccttgcaaatttacttcttcgttttctccgcttgtttgtgtctatgatcttgcctatgaaacatctttttatagtacacatatatgttgtcttattggaa
atatgatcggctaacatatttgttattgttattaacaaaaaaaataacatttgttattgttcaccggagttgatagtagcaatagtacatattacatg
gtcatatatgcatatggctacatgtttgagttatattcgattatctggtgaaagatgagattgtgagtttgtaagtttttgacagtgtagaggaggat
ccaaaagaaaagaggaaaaa**ATG**GACTATATGTATGGACCAGGGAGACACCATCTGTTTGTACCAGGACCAGTGAA
CATACCGGAACCGGTAATCCGGGCGATGAACCGGAACAACGAGGATTACCGGTCACCAGCCATTCCGGCGCTT
ACGAAAACATTGTTGGAGGATGTTAAGAAGATATTCAAGACCACATCAGGGACACCTTTTCTGTTTCCCACGAC
CGGGACTGGTGCTTGGGAGAGTGCCTTGACCAACACGTTATCTCCTGGAGACAGGATTGTTTCGTTTCTGATT
GGACAATTTAGCTTGCTCTGGATTGACCAGCAGAAGAGGCTTAATTTCAATGTTGATGTGGTTGAGAGTGATT
GGGGACAAGGTGCTAATCTCCAAGTCTTGGCCTCAAAGCTCTCACAAGACGAGAATCATACCATCAAAGCCAT
TTGCATTGTCCACAACGAGACCGCGACCGGAGTTACCAATGACATCTCTGCTGTCCGCACACTCCTCGgtcattaa
ttcctcacaaataatctgtttttcttttgtgttttctctgttttaaatctcacttacattagggtttacatacagATCACTACAAGCATCCGGCT
TTGCTGCTAGTGGACGGTGTTTCGTCCATCTGCGCGCTTGATTTCCGAATGGATGAGTGGGGAGTGGACGTGG
CCTTGACTGGGTCTCAGAAAGCCTTATCTCTTCCAACAGGACTTGGTATTGTCTGCGCCAGTCCTAAAGCTTTG
GAAGCTACCAAAACTTCTAAATCTCTCAAAGTATTCTTTGACTGGAATGACTACCTTAAGTTTTACAAGCTAGGA
ACCTATTGGCCATACACACCTTCCATTCAACTTCTCTACGGTCTTAGAGCTGCTCTTGATCTTATCTTTGAGGAA
GGACTTGAGAACATCATCGCCCGCCATGCTCGTTTGGGAAAGGCCACCAGgtaaatgaatcatataaaatgaatacactt
agtcctcttcgtaatcttgattcttactagtacattgactttggaaaacattatagctagagtttgattgttaatgatcatgtacatagGCTTGCG
GTGGAAGCATGGGGGCTGAAAAACTGCACACAGAAGGAGGAATGGATAAGTAACACAGTGACAGCAGTTAT
GGTGCCTCCGCATATAGACGGTTCGGAGATTGTGAGAAGGGCATGGCAGAGGTACAACTTAAGTCTTGGTCTT
GGTCTCAACAAAGTGGCTGGAAAGGTTTTCAGAATTGGACACCTAGGAAATGTGAATGAGgtacatatcgagattta
tcttagtgtaatccaattttcattgaccaaactcttatcaaacaatacaaatcgaacgcagTTGCAACTTCTCGGGTGTCTTGCGGGA
GTGGAGATGATACTGAAGGATGTTGGATACCCAGTTGTAATGGGAAGTGGAGTTGCAGCTGCCTCTACTTATC
TTCAGCACCACATTCCTCTCATTCCCTCTAGAATCTAAtccatgtggtccttctctttctttctcttcctctcaatgtaaacaaactctc
atgttttctcttttcttcatctatatttttactacattatctatgtttttatttgctatctccaacaaacttattgaacaaaagatcaaatatgtttattact
cactataaactttgtctcaacaacttcaacttatatgggtatgagggatgctcttacaatgttagtggaacataactcgagtctaaaactatgtttgg
gatgaggatactcgatcgtcaagtgaaaaaagataattttgatctctcatccatttatatggaggttggtttaaagacactatagtcgggagaagg
tgtcccatttcgcggtatgaagtcgaagacgactgagatataaggtctacatttggcggtatgaagtcgaagacgactgagatataaggtctaca
tttcgcggtatgaagtcgaagacgactgagatataaggtctacatttggcggtatgaatcaagaagacttatttcaagaggggtcttatctgtgac
aacaaaaggaaactcttgttgatgctttcttatgcctagtcagtaatcacaatgttctgcatatacaacctccatattaataagaaatattgaaattc
tttaaaatgtatttgcgtcttaacgcttttagtttaaac

Ananas comosus Agt1* (Aco003139) *genemodel extracted from PHYTOZOME v12.1

>Aco003139 | LG06:14066281..14071086 reverse
GGAGCAACGTTTCAAAGCAATACTACTGGACCCAGGAACCAACCAATAGGTACTCTCTCTCTCTCTCTCTCTC
ATAGAATTATAGTATCTGATTCTACATATATTGCTAGGAAAGTAGTGCATGCGATTTATGGAATTTGTTACGCA

AATTACTTGCTTTGTATTTAGCATACAAGCTATATTATGTTTGAAGGAGATGATCGTATGTTTCTTTTTGCATGTT
TTACTTCCGTAAGCATACATATGAAGCATCTCAAAAATAGACCACACATGTACACAAAAAAAAAAAAAACTACGC
TTCGTCTAATTATTTAGAAATGAACTCAAGTGAATATATAAAATTACTAAATTTCGAACTAATTAATCGAATATT
TAGTCAACTACTCTAAATACCATTGGTCACCCATGATAAAAGTGTTAATGTGAGATACTTAATTGAGCCTTTTAG
ATTAATCAGGTCACTCTTTCATCAAATTTTTTTTAAAAAAAATTACAGAAACTGCTAAAAGTAAGTGAGAGAATT
TCATAATTTGTTACTCGCACCAAAGTTTCAAACGTAGCAAGCGTACAGGGTGTGTAAAAGAAGTAAATGATTTA
AAACATGCGGCTTATAGAACATTAACAAGTCATTCATTTCTTAATTTATACACACACACACACTCTAAACAAA
AAAAAAAAAACATTCATTCATTTCTATATCCTGGTCGAGATAAAAAAATAAATTTTAAAAGTTCATCCTTATAGC
AAGAGATACAGGATATACCAAGGACGTTATTTTTAGGCACTAGCTTTCCCACGAGTAAGAACTTCTTTTTATTA
GATAAATTTTAACGGCTGAATATATCCGTGCATAAGATTTTTCTGAAATAACTCTTCTAAGCTTCATTTAGCAGC
TTCTGTAGTTCTGTTGTTCTAAATTTTTTTTTTTTTAATTGTTGTTTTTTTTTTGAGACCCTGGTTGTCTCACTTCTGT
AGCCTAGTTTATACATTCAATCAATGAAGTAGGTAGTAAGCTATCTTTTCTCAAAAAAAAACTCATCTAAGCTAA
CAACCAATTATTTTTTTAATAGTAGTTTGATAAATACAGTTGCCTCCAAATTGCACTTGATTTCATTTTTCGAAGA
TCGTCATCACAGCGACTCATTGTAAAAAAAAAAAAAAAAAAAAACACGTGGCGGCCATGACGCATCCGTCTCTT
TCCACTTTGCGTATAAGAGTACCTCACAAACGAAGGAAAAAAAGAGAGAGAGAGAGAGAGAGAGTGAGAGA
GAGTGAGAGAAAAAAAAAGTCCTCTTCTTTTTTTCTTCGGTACGAATGGAACGCTGGCCTTTTCCGTCTTACCCC
CATTAAATATTAGAAATTACACGCATCACTTACGCGCTTAAGCCACTCAATTGGTTCCATTTCCCATTTAATATCT
ATCTGGGGAAAAAAAAAAAAAAAAAAAACTTTGATTTTTAGGGACCATGTTCGAAAGTAGTATTTTCGGGGCCC
TAAAAGCAAGCAGGGTGTATTTTCTCACAGGTTAAGTTAAAAAAAAAAAAAAGAGAAAGAAAGAAAAGAAGAA
GAAGAAGAAGAAG**ATG**GATTATGTGTATGGACCTGGGAGAAACCACCTGTTCGTGCCGGGGCCGGTGAACAT
CCCCGAGCCGGTGATTCGGGCGATGAACCGCAATAACGAGGACTACCGATCTCCCGCCGTGCCCGCTCTGACA
AAGACGCTTCTCGAAGACGTCAAGAAGATCTTCAAGACAACATCCGCAACCCCCTTCGTAATCCCCACCACCGG
TACTTCCATCCATCCACNCAAAAAAAAAAAACTCTTCTAAGCTAACAACCAATTATTTGTTTTAATAGTAGTTTGA
TAAATACAGTTGCCTCCAAATTGCACTTGATTTCATTTTTCGAAGATCGTCATCACAGCGACTCATTGTAAAAAA
ACAAAAAAAAAAAAACACGTGGCGGCCATGACGCATCCGTCTCTTTCCACTTTGCGTATAAGAGGACCTCACAA
ACAAAGGAAAAAAGAGAGAGAGAGAGAGAGAGTGAGAGAAAAAAAAAGTCCTCTTCTTTTTCCTTCGGTACG
AATGGAACGCTGGCCTTTTCCGTCTTACCCCCATTAAATATTAGAAATTACACGTATCCGTCACGCGCTTAAGCC
ACTCAATTGGTTCCATTTCCCATTTAATATCTATCTGGGGAAAAAAAAAAAAAAAAAAACTTTGATTTTTAGGGAC
CATGTTCGAAAGTAGTATTTTCGGGGCCCTAAAAGCAAGCAGGGTGTATTTTCTCACAGGTTAAGTTAAAAAA
AAAAAAAAAGAGAAAGAAAGAAAAAAGAAGAAGAAGAAGATGGATTATGTGTATGGACCTGGGAGAAACCA
CCTGTTCGTGCCGGGGCCGGTGAACATCCCCGAGCCGGTGATTCGGGCGATGAACCGCAATAACGAGGACTA
CCGATCTCCCGCCGTGCCCGCTCTGACAAAGACGCTTCTCGAAGACGTCAAGAAGATCTTCAAGACAACATCCG
CAACCCCCTTCGTAATCCCCACCACCGGTACTTGCATCCATCCATCCATCCATCCACTCCAGTTACATCGGATCTT
GTGACAAGTCCAATATATAGTTCGTCACACGCTTTTTGCACCTCTAATTGTTTGTAACTATTTTTGTGTCCTGGAT
TAGGCACCGGAGCATGGGAAAGTGCGCTGACGAACACTCTATCACCTGGGGACAGAATCGTGTCCTTTCTGAT
CGGCCAATTCAGCCTCCTCTGGATCGACCAGCAGCAGCGCCTCAAGTTCGACGTGGACGTCGTCGAAAGCGAG
TGGGGCCGCGGCGCCAATCTCGACGTCCTGGAATCTAAACTGGCGGCCGACAGGAGTCACACCATCAAGGCT
ATCTGCATTGTTCACAACGAGACGGCCACCGGCGTCACCAACAATTTGCCGGCTGTGAGGAAACTGCTCGGTA
AGCGCGAACAATCGGAAAAAGCTTGGGGAGATGATTTTGTGTGCTTGTTGTTCTGTCTATATAATTTACAGCAG
TCGATAAGATGGTTTCGTAGAAAAATTATGCTTTTAAGCCTTCGATCCTACGCATCCAATTAAATAGGTAGATG
AATTCTCTGTTACTTGTTTATACTGTTATATTGGCATCAGATCAGGCATGGACTCTGTAATGCACCTATACATTG
TTTCAGCAGTAACGAAGGCTTAATTAGATGGTCGTAATATAGAAATAAGGTAGATGAATTATAGTCATCTTTAT
ATGCCTCTTCTTTGTATTCTAACATATTAAGTTACTTAATACGGCAGACGATTACAGCCATCCCGCACTAATACTT
GTTGATGGCGTGTCGTCGATATGTGCTCTTGACTTCCGAATGGACGAATGGGGG**ATTGATGTCGCCTTAACCG**
GCTCGCAGAAAGCTCTTTCGATGCCTACTGGAATGGGCATTCTTTGTGCGAGCCCGAAAGCTCTAGAAGCTT
CCAAAACAGCCAAGTCGGTCAGGGTTTTCTTCGACTGGAATGACTACTTGAAGTTCTACAAGCTCGGAACTT

ACTGGCCTTACACGCCTTCCATCCAACTACTTTACGGGCTAAGAGCAGCTTTGGATCTCATCTTTGAGGAGGG
GCTCGACAATGTCATCGAAAGGCATCGTCGCTTAGGAAAGGCCACAAGGTAACTAAGCACACAATCTTCTAT
CGAGTACTGCTGTAAAAGCTGGATTTTCATATATACATCTACTACTGAATAGCCGCTTTTTCGCCGCACTGTTG
GAATATGTTATAGATGGATATTACTTGGCACCGATTATTTTACGACCTCTTGTAACATAATCGGCGTAAAATG
ACCTTGCAGACTTGCTGTCGAAGCTTGGGGACTGAAGAATTGCACGCAGAAAGAGGAATGGTTCAGCGACA
CGGTCACTGCAGTTCTGGTGCCGCCTTACATTGATAGCGCTGAAATTGTGAGGAGGGCGTGGAAGAGATACA
ATCTAAGCTTGGGTTTGGGCCTCAACAAAGTGGCCGGAAAAGTGTTCAGGATAGGGCATCTCGGCAACCTCAA
TGAGGTATATTACAATTCTTGTTCATTTTCCGGGTTTTTCGACTTTCAAAAAAGAGAAGAAGATAAATAGAGAT
TATTTGCGTGCGGCCGACGGCCCAGAAGCCTCTTGTAAAACACCGTATTGTGCTGCTTCGCACAAAGGTGCCTC
TTAAAAAATAGGACCTCAATGCTTGCTTTAACTGTTAAACTGTTTTGTTAGTAAAACTCGATTGATACCGTCCAA
CGTCAAAGCTTTCTGTTTTTACACAGCTTATTTATGAATGAGCCAGATTTTGCAAGGCTACATGTAAATGGAACT
AAAATTTTTCCCAGCTTCTGCGCGCAAGTACTTGGCTAATAACAATGTAGAATCTTATGCATAATTTCAAGTATA
TAAGACTGATTTTTATCTATATTTTTCTGCAGTTGCAATTGCTGGGTTGCTTAAGTGGAGTAGAGATGGTGCTG
AAGGATGTGGGCTACCCAGTGAAGCTGGGGAGTGGAGTAGCTGCTGCAGCTGCTTATCTTCAGAACACTATCC
CTCTCATCCCCTCTCGAATCTGAGTTTTCTATAATTGCCTCCTTTGCTTCAAATCCTTGTAATTAAGGAAAAAAAA
AAATACTGTAATCTCATTCAGGAGGCTGAGGCCCCTGATAAATACTGTACTACTGTATCTAAATCTTTTACATTT
GAATATGGTAGTAGTTAAGCCCCTTCCAAAAAAAGCTTGTCACAAGTTAAAATGTTAGATTTACTCAAATCAG
